# Surprisingly Helpful: The Introduction of Portal Practice Slots to Address the Inbasket Explosion

**DOI:** 10.1007/s11606-025-09582-8

**Published:** 2025-05-29

**Authors:** Jennifer Reilly Lukela, James Henderson, Audrey Fan, Julia Chen, John Brinley, Joel Scharboneau, Eve A. Kerr

**Affiliations:** 1 Department of Internal Medicine, Michigan Medicine, University of Michigan, Ann Arbor, MI USA; 2 Center for Clinical Management Research, VA Ann Arbor Healthcare System, Ann Arbor, MI USA

**Keywords:** patient portal, inbasket, burnout, bell-being

## Abstract

**Background:**

Despite the dramatic increase in the volume of electronic health record (EHR) inbasket work, most organizations have not provided additional time to attend to this asynchronous care delivery.

**Aim:**

To improve physician well-being and reduce time spent in the EHR outside of work by providing structured time during the workday to address inbasket workload.

**Setting:**

An academic health system in the Midwest.

**Participants:**

Physicians in general medicine and medicine pediatrics with ≥ 1 half-day of clinic per week.

**Program Description:**

Implementation of one 20-min “portal practice slot” (PPS) in schedule templates per clinic half-day (4 h).

**Program Evaluation:**

After the intervention, a survey was distributed to assess physicians’ perceptions regarding the impact of PPS on workload and work-life balance. We also assessed for change in hours spent in the EHR on evenings and weekends (“pajama time”), relative value units (RVUs), and patient visits.

**Discussion:**

Despite no decrease in measured pajama time, most physicians reported that PPS improved their ability to complete asynchronous care and inbasket tasks in a timely manner during the workday. There was no significant change in the number of visits, visit hours, or RVUs generated. PPS appeared to be a successful tool to decrease the sense of “overwhelm” created by inbasket work and improve physician well-being.

**Supplementary Information:**

The online version contains supplementary material available at 10.1007/s11606-025-09582-8.

## INTRODUCTION

Primary care practice has changed significantly over the past decade. Studies have shown primary care physicians (PCPs) spend a significant amount of time outside of office visits in the electronic health record (EHR).^1–3^ While some of this EHR time involves traditional activities such as pre-visit chart review, documentation, medication refills, and results review, patients increasingly use the EHR’s portal to ask medical questions. As a result, there has been an explosion in care delivery outside of traditional office visits. Pre-pandemic, faculty at our institution estimated they spent an average of 20 hours weekly handling 390 inbasket tasks related to non-scheduled patient care.^3^ To handle this additional, largely uncompensated, work time, some faculty reduced their clinical effort to work part-time, taking a significant pay reduction to make the workload manageable and maintain work-life balance.^3^ During the COVID pandemic, the volume of asynchronous care further increased. Ambulatory physicians saw a 57% increase in inbasket volume,^4^ and this increase was felt most acutely by PCPs, who receive twice the volume of messages as other outpatient specialties.^5^ While there is evidence that PCP’s time in the inbasket may be stabilizing, it is now at a level substantially higher than it was pre-pandemic.^6,7^

There is also evidence demonstrating a link between inbasket burden and physician burnout, which was high pre-pandemic and is now at critical levels.^5,8,9^ This is likely a result of both the increased volume of inbasket messages and a perceived change in the expectation of the timeliness and urgency of a response.^10^ A recent study suggested a physician’s odds for burnout increased by 4% for each inbasket message received.^5^ Women physicians, who receive a higher volume of inbasket messages and shoulder a larger share of household responsibilities, may be at particular risk for burnout.^11–15^ Given high rates of burnout among the primary care workforce, the associated risks of continued physician attrition, and patients’ desire to get more care asynchronously, interventions to address how physicians integrate inbasket work into their workday are necessary.

Within this setting, we introduced “portal practice slots” (PPS) into our clinic template. The goal of this intervention was to reduce physicians’ time spent in the EHR outside of work by providing structured time to address inbasket workload and complete asynchronous patient care tasks during the workday. An overarching aim of this intervention was to improve physician well-being and sustainability of careers in academic outpatient general medicine.

## SETTING AND PARTICIPANTS

PCPs in general medicine at an academic health system in the Midwest.

## PROGRAM DESCRIPTION

In April 2022, we implemented one 20-minute PPS per half-day of clinic into schedule templates. For context, clinics have a 20/40 template with 20-minute urgent and chronic care visits and 40-minute preventive care and new patient visits. A full-time clinician spends 32 hours weekly in direct office-based clinical care. The PPS were intended to provide physicians dedicated time to attend to the inbasket during clinic.

## PROGRAM EVALUATION

### Methods

#### Design

We electronically surveyed all outpatient general internist faculty in April 2023, 12 months after the implementation of the intervention, to assess the impact of PPS. Using a pre-post design and a combination of EHR and administrative data for physicians who had established practices in the pre- and post-intervention periods, we assessed associations between PPS implementation and physician time in the EHR outside of work (“pajama time”), the number and total time of completed patient visits per physician, and relative value units (RVUs) per physician.

This quality improvement innovation was determined to be exempt by the University of Michigan Medical School Institutional Review Board.

#### Physician Survey

The survey included three questions addressing the impacts of PPS on workflow and well-being using a 5-point Likert scale. The survey was designed specifically to assess experiences with clinical care, including the inbasket, that we expected may be influenced by our portal practice quality improvement intervention. We summarized responses by reporting percentages of agreement (“agree” or “strongly agree”), neutrality (“neither agree nor disagree”) and disagreement (“disagree” or “strongly disagree”). Free-text responses were reviewed by three of the authors (JRL, JC, AF) to identify common themes. Additional questions captured information such as gender and clinical full-time equivalent (cFTE). The survey was distributed electronically via Qualtrics, with two email reminders sent to encourage completion. The survey is included in the supplementary online materials.

#### EHR-Based Summary Measures

We included physician months with ≥ 12.5% cFTE (1 half-day of clinic weekly) when analyzing EHR-based measures and excluded physicians with ≤ 6 months in either the pre- or post period. From these “established providers,” we excluded physicians hired since July 2021, since, due to a reduced clinic schedule during their onboarding, pre-post comparisons would be confounded by schedule template transitions. We obtained hours spent in the EHR outside of working hours (outside of 7am–5:30pm weekdays or any unscheduled time on a weekend) from Epic Signal. Since Epic does not report pajama time for providers with < 5 appointments per week, if a physician had a low-volume month below this threshold, we excluded that month from the analysis of pajama time. We extracted the number of completed visits, including face-to-face and virtual visits, hours of completed visits (visit hours), and RVUs from our health-system data warehouse.

We evaluated associations between PPS and pajama time, visits, visit hours, and RVUs using monthly, physician-level summaries from April 2021 to September 2023. The 12-month pre-period was April 2021 to March 2022, and the 18-month post period was April 2022 to September 2023. We normalized all measures by cFTE, excluding time for resident supervision, with 1.0 cFTE representing 32 clinic and 8 administrative hours. To assess associations with PPS, we modeled each EHR measure using an interrupted-time series regression implemented using generalized linear mixed models with physician-level random intercepts and trends (see supplementary methods). We summarized associations between measures and PPS using Average Marginal Effects (AME) representing the average differences between the expected value of each measure in the post-period and a counterfactual prediction made by setting the post-PPS indicator to zero.

We computed ratios of 20- to 40-minute visits before and after PPS to ensure visit rates were not confounded by this ratio. We also performed a sensitivity analysis limiting to physician months with ≥ 0.67 cFTE to assess whether results were different for those with a greater proportion of clinical work.

### Survey Results

We distributed the post-intervention survey to 102 general medicine faculty physicians, of whom 68 (67%) were female, 47 (61%) had a cFTE ≥ 0.75, and 74 (73%) responded. Consistent with our faculty demographics, 51 (69%) of 74 respondents identified as female and 43 (58%) reported cFTE ≥ 0.75.

Among responders, PPS was well received, with 65 (88%) agreeing PPS decreased the amount of time spent on patient care outside of regular working hours, 5 (7%) neutral, and 4 (5%) disagreeing. Asked if PPS improved their ability to address urgent inbasket messages during work hours, 69 (93%) agreed, 2 (3%) responded neutrally, and 3 (4%) disagreed. Similarly, 64 (86%) agreed PPS made inbasket work feel less overwhelming, while 6 (8%) were neutral and 4 (5%) disagreed.

Review of qualitative comments identified several themes, including improved ability to address urgent inbasket issues, improved flexibility to “catch up” in clinic, enhanced ability to overbook patients in clinic if needed, the perception of decreased work outside of clinic, concern regarding decreased patient access to appointments, the sense that the time allotted for PPS was insufficient, and a decrease in the sense of feeling overwhelmed (Table 1). An overall common theme from qualitative survey comments was that PPS proved to be “surprisingly helpful.”

**Table 1:** Common Themes Identified in Qualitative Comments

Qualitative comment themes	*n* of survey respondents addressing (*N* = 62)	Sample quotes
Improved ability to address urgent inbasket messages	32	• “(These are helpful) predominantly… to address urgent messages (phone or portal) that should not wait until end of a session or end of day; this is extremely important for both safety and quality of care for our patients” • “They have allowed me to address urgent issues in a timely fashion”
Allow time to “catch up”	14	• “These slots are an important ‘release valve’ of the stress of clinical care. Sometimes I don’t get to use them for inbasket catch-up if I’m running behind with scheduled patient visits. But even then, it feels like a more-than-20-minute improvement in my life.”
Perception of less pajama time	12	• “(PPS) have been extremely helpful in improving the amount of work I am able to achieve within business hours” • “Less pajama time for sure!!!”
“Not enough”/still too much inbasket work volume	10	• “It does not fully account for the amount of inbox time we need to spend each day.” • “We all know the volume is tremendously higher and I would advocate that MORE time is indicated to cover this workload” • “Workload has gone from entirely crushing to simply overwhelming”
Enhanced ability to overbook/add on patients with urgent concerns	10	• “(PPS) also allows me to have the option to add patients on when my schedule is otherwise full.”
Decreased stress/burnout	5	• “They have decreased feelings of burned out and overwhelm” • “They make a huge difference in how harried I feel”
Negative impact on access	3	• “(PPS) have provided time but have also made the overwhelming problem of patient access worse”
Make maintaining higher FTE possible	2	• “I cannot express strongly enough, as someone who is a 1.0 cFTE, how significantly the portal practice time has helped me manage my clinical duties... It is THE reason I feel I can continue at my current FTE”

### EHR Results

We included 2079 provider months, including 842 and 1237 in the pre- and post periods, respectively, representing 71 established physicians from 11 clinics in the EHR-based analyses. These 71 physicians included 50 (70%) women and 37 (52%) with a median cFTE ≥ 0.75.

Analysis of “Pajama Time” included 2037 provider months from 70 providers. There was no change in “Pajama Time” associated with the introduction of PPS (Table 2 and Table S1) although a sensitivity analysis that examined physicians with higher cFTE suggested larger, though not statistically significant, decreases in Pajama Time (Table S2).

**Table 2:** Association Between PPS and Pajama Time, RVUs, and Visits

Measure	Pre^1^ PPS Mean^2^ (95% CI)	Post^3^ — expected^4^ without PPS Mean (95% CI)	Post — observed with PPS Mean (95% CI)	Average marginal effect^5^ (95% CI)
Pajama Time^6^, hours/cFTE/month	22.5 (17.8 to 27.2)	21.3 (15.6 to 26.9)	21.5 (16.8 to 26.2)	0.3 (−3.1 to 3.6)
Relative Value Units (RVU)^7^, RVU/cFTE/month	360.6 (311.9 to 409.3)	358.6 (304.6 to 412.7)	377.5 (332.3 to 422.6)	18.8 (−14.9 to 52.5)
Completed visits, visits/cFTE/month	218.2 (208.6 to 227.8)	224.9 (203.8 to 246.1)	212.3 (201.6 to 223.0)	−12.6 (−30.8 to 5.6)
Completed visit hours, hours/cFTE/month	101.8 (96.8 to 106.7)	104.9 (94.8 to 114.9)	98.5 (93.7 to 103.4)	−6.3 (−15.1 to 2.5)

*PPS*, portal practice slots; *cFTE*, clinical full-time equivalent

^1^April 2021 to March 2022 (12 months)

^2^All means are weighted by cFTE

^3^April 2022 to September 2023 (18 months)

^4^The “post — expected” column represents a counterfactual mean computed by averaging predictions in the post period with the post-PPS variable set to zero

^5^The average marginal effect is the mean unit-level difference between observed and expected predictions, averaged over all provider months in the post period. Each AME is reported with a 95% confidence interval (CI). CIs that exclude 0 would be statistically significant

^6^Uses the “pajama time” numerator from Epic Signal but normalized using cFTE, excluding resident supervision. A cFTE of 1.0 = 32 clinic hours and 8 administrative hours

^7^We are reporting RVUs for which the physician is the service provider

We found no significant reductions in visits or visit hours per cFTE per month (Table 2). The ratio of 20-to-40-min visits also did not change with the introduction of PPS (1.29 pre-PPS and 1.30 post-PPS). Similarly, we did not find a significant association between PPS and RVUs (Table 2).

## DISCUSSION

In this innovation, structured 20-minute portal practice slots were inserted into clinic templates to provide physicians time to address inbasket tasks and deliver asynchronous care during the workday. Our innovation was extremely well received by outpatient general internists. It contributed to a decreased sense of feeling overwhelmed by the inbasket, along with a perception of improved ability to address urgent asynchronous care issues in a timely fashion. Physicians also believed that PPS decreased the amount of work they needed to complete outside of usual working hours.

Interestingly, formal assessment of physician “pajama time” did not change significantly. There are several possible explanations for this seeming disconnect between perception and measured reality. In our qualitative survey, physicians commented on how the structured time provided by PPS allowed them to attend to urgent portal and phone messages and that this improved their well-being by decreasing the constant worry that something urgent would be “missed.” Physicians also commented on how much they appreciated having control over a small amount of time in their workday. Past work has identified how “agency in the workplace” is a key element to physician well-being and that transition away from autonomy in scheduling may be exacerbating burnout.^16,17^ In addition, several physicians commented on how much they appreciated that PPS was a system-level acknowledgement of the significant increase in inbasket work, even while acknowledging that 20 minutes per half day does not address the volume of asynchronous work. It may be that these perceived benefits resulted in improved overall well-being and resulted in the *impression* of a reduced after-hours workload, even though the overall workload did not change substantially.

There were costs associated with the implementation of PPS. Given that the implementation of the PPS reduced one visit slot per half-day, we anticipated that there would be a reduction in total visit hours and patient access for face-to-face care, with visit hours declining by as much as 8.3%. While there was a trend toward reduced visits and visit hours, these were not statistically significant, and the point estimate suggests a smaller reduction than anticipated. This is likely due in part to physicians sometimes using PPS to overbook patients with urgent complaints. Additionally, while we anticipated a concurrent reduction in RVUs per cFTE, this was not observed.

There are limitations to our analysis of this innovation. The physician survey used to assess physician experience did not contain validated instruments. Additionally, we used pajama time in our analysis, which only looks at work in the EHR outside of 7am–5:30pm on weekdays and non-scheduled weekend time. There are concerns that the time estimates captured by EPIC Signal data may underestimate total time spent, given that you must engage with the keyboard every 5 seconds for work time to be captured.^6^ Moreover, for physicians who reduced their hours during the study, normalizing EHR measures, including pajama time, by cFTE may be inadequate, given that their panel size, i.e., the number of patients they are responsible for, was not immediately reduced proportionally. Additionally, this was a single institution’s experience and given the relatively small number of physicians and variability of the metrics used, we lacked power to see small differences, evidenced by wide confidence intervals on the intervention effects. Finally, without a control group, we cannot account for other contemporaneously occurring operational changes, such as improvements in billing practices, including for portal messages.

Despite these limitations, this innovation appeared to be successful in addressing our primary aim of improved physician well-being and a decrease in the sense of “overwhelm” created by inbasket work. This improvement occurred despite no significant reduction in the Epic measure of pajama time and with less negative impact on visit number or revenue than anticipated. These findings support the concept that system-level change recognizing inbasket work is possible and necessary to meaningfully address this issue. While portal practice slots by no means “fixed the problem,” they appear to have had a positive and meaningful impact at our institution.

## Supplementary Information

Below is the link to the electronic supplementary material.

Supplementary file1 (DOCX 27 KB)
