# Supplementary material for: Surprisingly Helpful: The Introduction of Portal Practice Slots to Address the Inbasket Explosion
Source: J Gen Intern Med. 2025 May 29;40(10):2445–9. doi: 10.1007/s11606-025-09582-8 (PMC12343403; doi:10.1007/s11606-025-09582-8)
Supplement: Supplementary file 1 — Supplementary file1 (DOCX 27 KB) [file 11606_2025_9582_MOESM1_ESM.docx]

**Table of Contents**

Supplemental Methods …………………………………………………………………… Page 2

Physician Survey …………………………………………………………………………. Page 3

Supplementary Tables ……………………………………………………………………. Page 4

**Supplemental Methods**

To assess associations with PPS, we modeled each EHR measure using an interrupted-time series regression implemented using generalized linear mixed models with physician level multivariate Normal random intercepts and trends. Each model included a binary indicator for the post PPS period, a time trend representing the number of months before or after the introduction of PPS in April 2022, and an interaction allowing this trend to differ between the pre and post periods. Models also all included indicator variables for the month of year to control for potential seasonality. We modeled “Pajama Time”, visit hours, and RVUs with a Gamma family and log link, and visits with a Negative Binomial family and log link.

We summarized the association between each measure and PPS using the Average Marginal Effect (AME) for the post period indicator averaged over provider months in the post period. These AMEs represent the average difference, on the scale of each measure, between the expected value of the measure in the post period and a counterfactual prediction made by setting the post-PPS indicator to zero. Statistical analyses were done in R v4.3.0 using the “lme4”, “MASS”, and “marginaleffects” packages.

**Physician Survey**

*Distributed in April 2023* *via email on Qualtrics platform*

Q1 - The portal practice slots have decreased the amount of time I spend on patient care activities outside of my regular working hours (which includes administrative time)

Q2 - The portal practice slots have improved my ability to address urgent inbasket

messages during working hours

Q3 - The portal practice slots have made inbasket related work feel less overwhelming

*Questions 1-3 allow response using 5-point Likert scale response (strongly agree, agree, neither agree or disagree, disagree, strongly disagree)*

Q5 - Please provide your current cFTE (time in clinic)

<0.25, 0.25-0.49, 0.5-0.74, 0.75-0.99, 1.0

Q6 - Please specify your gender

Male, Female, Non-binary/third gender, prefer not to say

Q7 - Please comment on how the 20-minute portal practice slots have or have not been helpful in your care for patients, appointment access, and managing your work and personal time.

**Supplementary Tables**

**Table S1.** *Sample size and parameter estimates from regression models for each outcome measure.*

|  | **Estimate (95% CI)** | | | |
| --- | --- | --- | --- | --- |
| **Parameter** | **“Pajama Time”** | **RVU** | **Visit Hours** | **Visits** |
| *Parameter Estimates* | | | | |
| Intercept^1^ | 2.89  (2.66-3.11) | 5.84  (5.77-5.90) | 4.64  (4.59-4.69) | 5.38  (5.32-5.44) |
| Post Intervention | 0.08  (-0.01-0.17) | 0.02  (-0.03-0.08) | -0.01  (-0.06-0.04) | -0.02  (-0.06-0.03) |
| Trend (months) | -0.00  (-0.01-0.01) | 0.00  (-0.00-0.01) | 0.00  (-0.00-0.01) | 0.00  (-0.00-0.01) |
| Post Intervention x Trend | -0.01  (-0.02-0.00) | 0.00  (-0.00-0.01) | -0.00  (-0.01-0.00) | -0.00  (-0.01 to 0.00) |
| February | -0.11  (-0.20 to -0.02) | -0.10  (-0.15 to -0.04) | -0.09  (-0.13 to -0.04) | -0.10  (-0.14 to -0.05) |
| March | -0.14  (-0.24 to -0.05) | -0.01  (-0.07-0.05) | -0.01  (-0.06-0.04) | -0.02  (-0.07-0.03) |
| April | -0.03  (-0.12 to 0.06) | -0.10  (-0.15 to -0.04) | -0.10  (-0.15 to -0.05) | -0.10  (-0.14 to -0.05) |
| May | -0.09  (-0.19 to -0.00) | -0.12  (-0.17 to -0.06) | -0.11  (-0.16 to -0.06) | -0.11  (-0.15 to -0.06) |
| June | -0.23  (-0.32 to -0.14) | -0.15  (-0.21 to -0.10) | -0.16  (-0.21 to -0.11) | -0.16  (-0.20 to -0.11) |
| July | 0.03  (-0.05-0.12) | -0.10  (-0.15 to -0.04) | -0.13  (-0.18 to -0.08) | -0.12  (-0.17 to -0.07) |
| August | -0.18  (-0.27 to -0.10) | 0.03  (-0.03-0.08) | 0.01  (-0.03-0.06) | 0.01  (-0.04 to 0.05) |
| November | -0.08  (-0.17-0.01) | -0.02  (-0.08-0.04) | -0.03  (-0.07-0.02) | -0.03  (-0.08 to 0.01) |
| December | 0.03  (-0.06-0.13) | -0.06  (-0.12 to -0.01) | -0.09  (-0.14 to -0.04) | -0.08  (-0.14 to -0.04) |
| *Physician-Level Random Effects* | | | | |
| Intercept, SD | 0.38 | 0.17 | 0.09 | 0.18 |
| Trend, SD | 0.01 | 0.01 | 0.01 | 0.01 |
| Intercept-Trend Correlation | 0.02 | -0.25 | 0.16 | 0.18 |
| Residual, SD | 0.39 | 0.23 | 0.20 | - |
| Theta^2^ | - | - | - | 33.6 |

CI = Confidence Interval, RVU = Relative Value Unit, SD = Standard Deviation

^1^The first month of the intervention (April 2022) is time 0; January is the reference month.

^2^Theta is the dispersion parameter for the negative binomial model. The higher theta is, the closer the distribution is to Poisson.

**Table S2.** *Results from sensitivity analysis limiting to months in which providers had > 0.67 cFTE.* This sensitivity analysis utilized 48 providers and 1,083 provider months.

| **Measure** | **Pre^1^ PPS,**  **Mean^2^ (95% CI)** | **Post^3^ – Expected^4^ without PPS,**  **Mean (95% CI)** | **Post – Observed with PPS,**  **Mean (95% CI)** | **Average Marginal Effect^5^,**  **(95% CI)** |
| --- | --- | --- | --- | --- |
| Pajama Time^6^,  hours / cFTE / month | 19.6  (14.2-25.0) | 20.1  (12.8-27.4) | 18.4  (13.1-23.8) | -1.6  (-6.2 to 2.9) |
| Relative Value Units (RVU)^7^, RVU / cFTE / month | 327.4  (267.7-387.0) | 341.3  (271.6-410.9) | 342.1  (283.0-401.2) | 0.9  (-39.5 to 41.2) |
| Completed visits, visits / cFTE / month | 196.2  (185.4-207.0) | 217.3  (193.0-241.6) | 193.3  (183.8-202.9) | -24.0  (-46.4 to -1.6) |
| Completed visit hours, hours / cFTE / month | 94.3  (88.8-99.8) | 101.7  (89.8-113.6) | 92.1  (86.4-97.8) | -9.6  (-201 to 0.9) |

PPS = Portal Practice Slots, cFTE = clinical full time equivalent

^1^April 2021 to March 2022 (12 months).

^2^ April 2022 to September 2023 (18 months).

^3^All means are weighted by cFTE.

^4^The “Post – Expected” column represents a counterfactual mean computed by averaging predictions in the post period with the post-PPS variable set to zero.

^5^The average marginal effect is the mean unit-level difference between observed and expected predictions, averaged over all provider months in the post period. Each AME is reported with a 95% confidence interval (CI). CIs that exclude 0 would be statistically significant.

^6^Uses the “Pajama Time” numerator from Epic Signal but normalized using cFTE excluding resident supervision. A cFTE of 1.0 = 32 clinic hours and 8 administrative hours.

^7^We are reporting RVUs for which the physician is the service provider.
